# Assessment of SARS-CoV-2 Transmission on an International Flight and Among a Tourist Group

**DOI:** 10.1001/jamanetworkopen.2020.18044

**Published:** 2020-08-18

**Authors:** Sebastian Hoehl, Onur Karaca, Niko Kohmer, Sandra Westhaus, Jürgen Graf, Udo Goetsch, Sandra Ciesek

**Affiliations:** 1 Institute for Medical Virology, University Hospital, Goethe University Frankfurt am Main, Frankfurt am Main, Germany; 2 University Hospital Frankfurt am Main, Frankfurt am Main, Germany; 3 Health Protection Authority, City of Frankfurt, Frankfurt am Main, Germany; 4 Fraunhofer Institute for Molecular Biology and Applied Ecology, Branch Translational Medicine und Pharmacology, Frankfurt, Frankfurt am Main, Germany; 5 German Centre for Infection Research, Deutsches Zentrum für Infektionsforschung, External Partner Site Frankfurt, Frankfurt am Main, Germany

## Abstract

This case series describes severe acute respiratory syndrome coronavirus (SARS-CoV-2) transmission on a international commercial airline flight.

## Introduction

This case series assessed a commercial airline flight from Tel Aviv, Israel, to Frankfurt, Germany, that occurred on March 9th, 2020. Among 102 passengers on a Boeing 737-900 aircraft were 24 members of a tourist group. Starting 7 days earlier, the group had contact with a hotel manager who later received a diagnosis of coronavirus disease 2019 (COVID-19). No member of the group had received a diagnosis of COVID-19 before the flight, and no measures to prevent transmission (eg, wearing of masks) had been applied. The flight duration was 4 hours 40 minutes.

## Methods

At the destination airport, we conducted a medical evaluation of the tourist group, including testing for severe acute respiratory syndrome coronavirus (SARS-CoV-2) in a throat swab specimen. In addition, we contacted all passengers 4 to 5 weeks later by phone and conducted structured interviews. Passengers were asked whether they had contact with a person with COVID-19. They were prompted to report symptoms and asked about previous testing for SARS-CoV-2. A semiquantitative SARS-CoV-2 IgG antibody test (EUROIMMUN) was offered to all passengers who had been seated within 2 rows of the index cases and to those who reported to have been symptomatic. Borderline and positive results in the IgG test were confirmed with a plaque reduction neutralization test (PRNT). Oral informed consent was obtained from all study participants, and additional written consent was obtained for laboratory tests. This study was exempt from a formal ethics committee approval by the University Hospital Frankfurt, Goethe University, Frankfurt, Germany.

## Results

**Figure 1.**
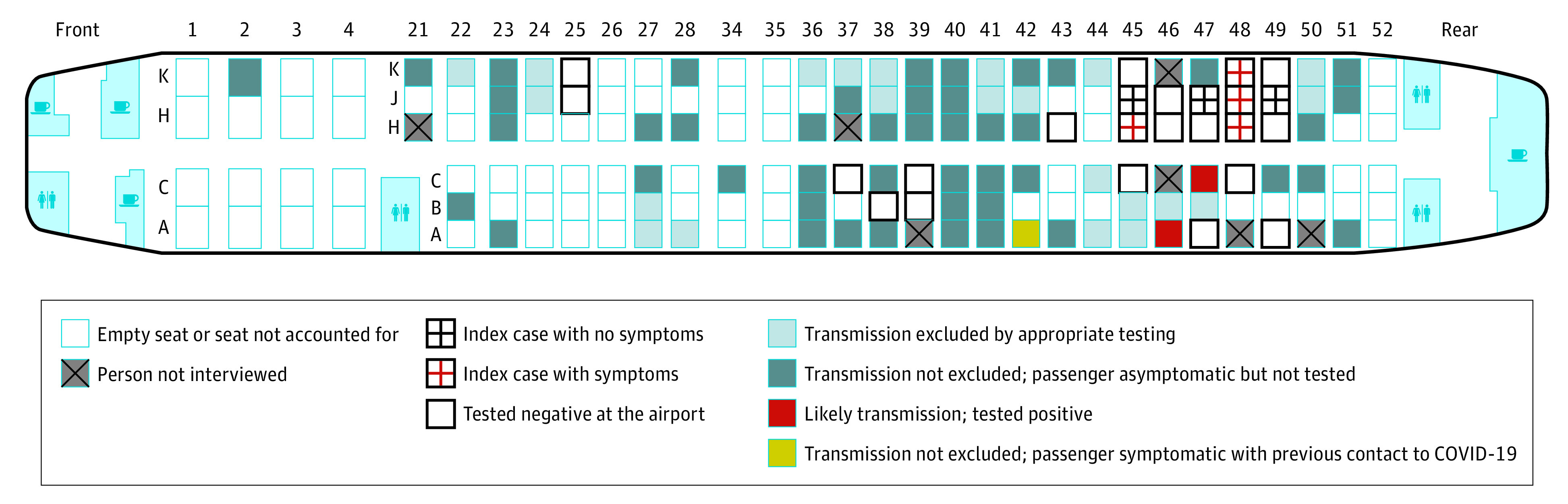
Seating of the Index Cases and Other Passengers on the Aircraft (Boeing 737-900) COVID-19 indicates coronavirus disease 2019.

Of the 24 members of the tourist group, 7 tested positive for SARS-CoV-2 RNA in a throat swab sample on arrival. Four of the 7 were symptomatic during the flight, 2 were presymptomatic, and 1 remained asymptomatic (Figure 1).

**Figure 2.**
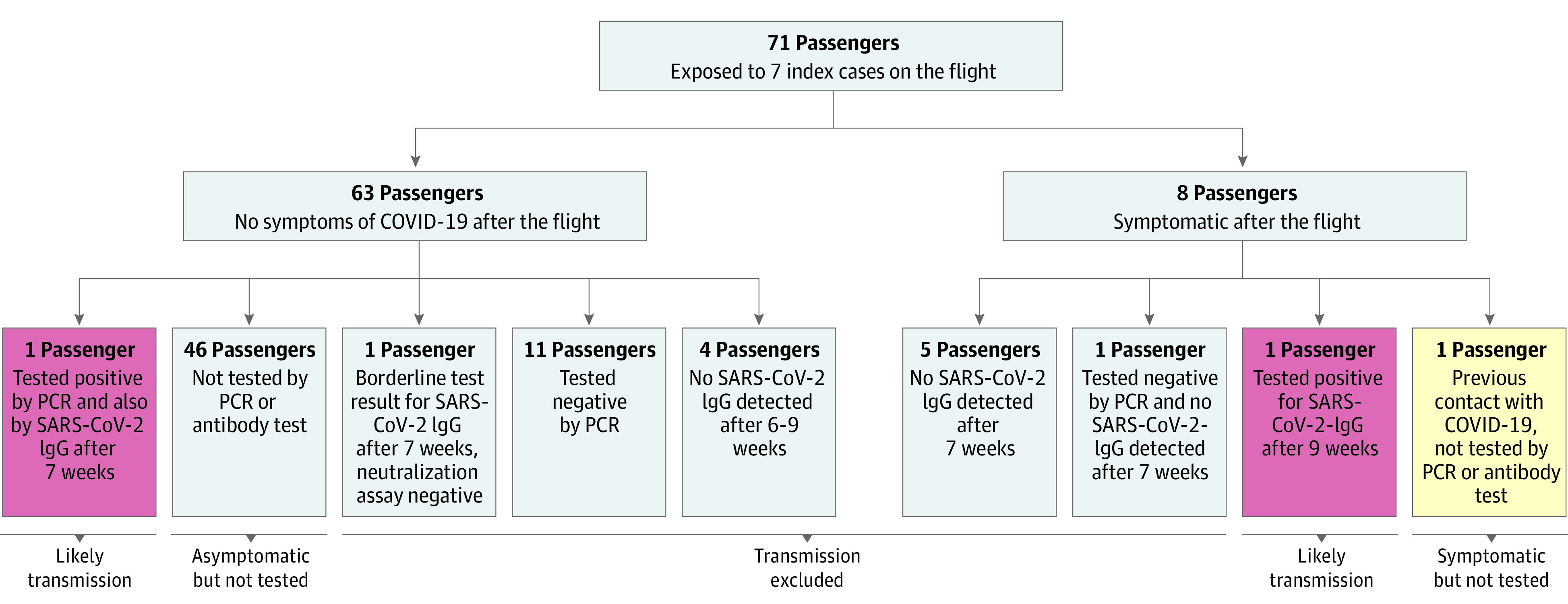
Flowchart of the Tests for Severe Acute Respiratory Syndrome Coronavirus (SARS-CoV-2) and of Symptoms of the 71 Passengers Who Were Interviewed COVID-19 indicates coronavirus disease 2019; PCR, polymerase chain reaction.

A total of 71 of the other 78 passengers (91%) who had been exposed to the group on the flight completed the interview. Serum samples were obtained from 13 of these individuals 6 to 9 weeks after the flight (Figure 2). One reported having tested positive by polymerase chain reaction 4 days after the flight. This passenger did not recall any symptoms. We detected SARS-CoV-2 IgG 7 weeks after the flight, and the PRNT result was also positive. The passenger negated contact with patients with COVID-19 before or after the flight.

Seven other passengers reported having had symptoms suggestive of COVID-19 within 14 days after the flight. One had a headache, muscle ache, and hoarseness starting 5 days after the flight. This passenger had not been tested and negated known contact with a patients with COVID-19. The passenger was in quarantine for 14 days starting 1 day after the flight. We obtained a serum sample 9 weeks after the flight and detected SARS-CoV-2 IgG. The PRNT had a borderline result.

We also obtained serum samples from 6 other symptomatic and 5 asymptomatic passengers 6 to 9 weeks after the flight. All tested negative except for 1, who had a borderline result on the SARS-CoV-2 IgG test but had a negative result on the PRNT. SARS-CoV-2 transmission during the flight was not excluded for 1 symptomatic passenger with previous contact with a patients with COVID-19 and 46 asymptomatic passengers who were not tested.

## Discussion

We discovered 2 likely SARS-CoV-2 transmissions on this flight, with 7 index cases. These transmissions may have also occurred before or after the flight. The risk of transmission of droplet-mediated infections on an aircraft depends on proximity to an index case and on other factors, such as movement of passengers and crew, fomites, and contact among passengers in the departure gate.^1^ In our study, both passengers with likely onboard transmission were seated within 2 rows of an index case.

The airflow in the cabin from the ceiling to the floor and from the front to the rear may have been associated with a reduced transmission rate.^2^ It could be speculated that the rate may have been reduced further had the passengers worn masks.

It has previously been observed for SARS and influenza that transmission may also occur among passengers seated beyond the 2-row perimeter,^2,3^ indicating possible airborne transmission. Our findings do not rule out airborne transmission of SARS-CoV-2 in an airplane cabin.

This study had several limitations. We did not obtain information on the crew of the airplane and were not able to contact all passengers. We also did not obtain antibody tests from all passengers. Additional transmissions may have occurred and remained undetected.
