# Error in the Affiliations

**DOI:** 10.1001/jamanetworkopen.2020.22333

**Published:** 2020-09-16

In the Research Letter, “Assessment of SARS-CoV-2 Transmission on an International Flight and Among a Tourist Group,”^1^ published on August 18, 2020, the affiliations were incorrect for Sandra Ciesek, MD. They should have been Institute for Medical Virology, University Hospital, Goethe University Frankfurt am Main, Frankfurt am Main, Germany; Fraunhofer Institute for Molecular Biology and Applied Ecology, Branch Translational Medicine und Pharmacology, Frankfurt, Frankfurt am Main, Germany; and German Centre for Infection Research, Deutsches Zentrum für Infektionsforschung, External Partner Site Frankfurt, Frankfurt am Main, Germany. This article has been corrected.

## References

[1] Hoehl S, Karaca O, Kohmer N, Assessment of SARS-CoV-2 transmission on an international flight and among a tourist group. JAMA Netw Open. 2020;3(8):e2018044. doi:10.1001/jamanetworkopen.2020.18044 PMC7435338 32809029

